# A Novel Genetically Encoded Single Use Sensory Cellular Test System Measures Bicarbonate Concentration Changes in Living Cells

**DOI:** 10.3390/s20061570

**Published:** 2020-03-11

**Authors:** Kevin Bernhard, Cordula Stahl, Regina Martens, Manfred Frey

**Affiliations:** Steinbeis-Innovationszentrum Zellkulturtechnik, c/o University of Applied Sciences Mannheim, Paul-Wittsack-Str.10, D-68163 Mannheim, Germany; kevin.bernhard@stz-frey.de (K.B.); c.stahl@stz-frey.de (C.S.); regina.martens@stz-frey.de (R.M.)

**Keywords:** bicarbonate, single use sensory cellular test system, anionophore, cystic fibrosis transmembrane conductance regulator (CFTR), membrane transport, adenylate cyclase (adenylyl cyclase), Förster resonance energy transfer (FRET), molecular imaging

## Abstract

Bicarbonate plays a central role in human physiology from cellular respiration to pH homeostasis. However, so far, the measurement of bicarbonate concentration changes in living cells has only been possible by measuring intracellular pH changes. In this article, we report the development of a genetically encoded pH-independent fluorescence-based single-use sensory cellular test system for monitoring intracellular bicarbonate concentration changes in living cells. We describe the usefulness of the developed biosensor in characterizing the bicarbonate transport activities of anionophores—small molecules capable of facilitating the membrane permeation of this anion. We also demonstrate the ability of the bicarbonate sensory cellular test system to measure intracellular bicarbonate concentration changes in response to activation and specific inhibition of wild-type human CFTR protein when co-expressed with the bicarbonate sensing and reporting units in living cells. A valuable benefit of the bicarbonate sensory cellular test system could be the screening of novel anionophore library compounds for bicarbonate transport activity with efficiencies close to the natural anion channel CFTR, which is not functional in the respiratory epithelia of cystic fibrosis patients.

## 1. Introduction

**Figure 1:**
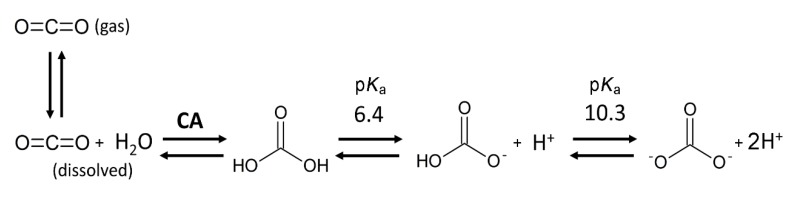
pH-dependent equilibria of bicarbonate. Bicarbonate (HCO_3_^−^) is in pH-dependent equilibria with carbonate (CO_3_^2−^) and carbonic acid (H_2_CO_3_). Carbonic acid can be converted to water and CO_2_ by the enzyme carbonic anhydrase (CA). At air interfaces of the aqueous solution, dissolved CO_2_ is in equilibrium with gaseous CO_2_.

Bicarbonate plays a central role in human physiology from cellular respiration to pH homeostasis. Regulation of bicarbonate transport across cell membranes is therefore of critical importance. Bicarbonate is a labile molecule involved in several pH-dependent equilibria (Figure 1). At air–liquid interfaces, as in the lungs, gaseous CO_2_ is in equilibrium with dissolved CO_2_. The enzyme carbonic anhydrase (CA) catalyzes the reversible reaction of water and CO_2_ to form carbonic acid, which in turn is in equilibrium with bicarbonate. CA is a ubiquitous enzyme found in nearly all organisms. It catalyzes the rapid conversion of carbon dioxide produced by cellular respiration to bicarbonate in all tissues. In contrast to CO_2_, which can diffuse across biological membranes, bicarbonate does not permeate cell membranes but instead requires bicarbonate transport proteins for transmembrane movement [1].

Bicarbonate is the natural buffer system in living cells; therefore, bicarbonate transport across biological membranes affects the intracellular pH. Bicarbonate influx into a cell increases the intracellular pH; accordingly, bicarbonate efflux out of the cell decreases the intracellular pH. Bicarbonate concentration changes inside a cell result from an interplay between different bicarbonate transporters, ion channels, cytosolic and extracellular carbonic anhydrase enzymes, and pH changes. Part of these complex interactions is the bicarbonate transport metabolon, a complex composed of bicarbonate transporters and cytosolic and extracellular carbonic anhydrase enzymes [2].

Since the intracellular pH needs to be tightly regulated in order for homeostasis to be maintained, pH changes inside a cell trigger cellular responses resulting in the compensation of these pH changes through Na^+^/H^+^ exchangers, passive proton conductance channels, and voltage-gated proton channels [3].

In humans, bicarbonate transport proteins, metal transporters, and anion channels contribute to the movement of bicarbonate across membranes. Bicarbonate transporters are involved in cell volume regulation and contribute to the removal of respiratory CO_2_. Defective bicarbonate transport leads to various diseases including brain dysfunction [4], kidney stones [5], systemic acidosis [6], and hypertension [7]. Altered expression levels of bicarbonate transporters in cancer patients suggest an important role of these transport proteins in cancer; indeed, pH dysregulation is a hallmark of cancer [8]. Moreover, the rare genetic disease cystic fibrosis (CF) is caused by defects in the anion-selective channel protein cystic fibrosis transmembrane conductance regulator (CFTR) due to mutations in the CFTR-encoding gene. In healthy individuals, CFTR functions as a transmembrane channel protein selective for chloride and bicarbonate in the apical membrane of epithelia. To date, more than 2000 mutations in the CFTR-encoding gene are known and are grouped in six classes according to the respective mutation. Mutations in the CFTR-encoding gene can cause the complete absence of CFTR protein synthesis, impairments in protein trafficking and folding, or non-functional proteins. The pharmaceutical company Vertex has developed the CFTR corrector lumacaftor and the CFTR potentiator ivacaftor which have been approved for CF therapy. However, lumacaftor and ivacaftor only possess therapeutic value for a very limited number of mutations in the CFTR-encoding gene, and, consequently, only very few CF patients benefit from them. In contrast, replacing the defective CFTR activity with anionophores would be a novel therapeutic approach for the treatment of CF that is independent of the mutation the patient harbors and, thus, would have a clear advantage over CF therapy using correctors and potentiators of CFTR [9,10,11].

Despite the paramount importance of bicarbonate and bicarbonate transport at the cellular level, there is no method known to us that does not depend on the measurement of intracellular pH changes for monitoring bicarbonate concentration changes in living cells.

In this article, we report the development of a genetically encoded single-use fluorescence-based sensory cellular test system for the measurement of intracellular bicarbonate concentration changes in living cells in a pH-independent manner. Upon induction of transgene expression, the sensory cellular test system can be used for a limited period of time, since longer expression of the recombinant sensor proteins results in severe cell stress and subsequent cell death. The sensory cellular test system is particularly useful for studying the functionality of bicarbonate transport proteins when co-expressed with the sensing and reporting units. We demonstrate the ability of the bicarbonate sensory cellular test system to measure bicarbonate concentration changes in Chinese hamster ovary (CHO) cells mediated by the naturally occurring anionophore prodigiosin. Further, we demonstrate the ability of the bicarbonate sensory cellular test system to measure intracellular bicarbonate concentration changes mediated by activation and specific inhibition of co-expressed wild-type human bicarbonate-conducting CFTR protein.

## 2. Materials and Methods

### 2.1. Cell Culture

CHO-K1 cells (DSMZ-No. ACC-110) were cultivated in an incubator in 5 % CO_2_ atmosphere and at 37 °C in Ham’s F12 medium (Sigma-Aldrich, Steinheim, Germany), supplemented with 10 % Gibco™ fetal bovine serum (Thermo Fisher, Darmstadt, Germany), 100U/ml penicillin (Sigma-Aldrich) and 100 mg/ml streptomycin (Sigma-Aldrich, Steinheim, Germany).

### 2.2. Generation of the sAC-CEPAC Bicarbonate Sensory Cellular Test System

The genetically encoded fluorescent CEPAC cAMP FRET reporter (cAMP-detecting FRET biosensor mCerulean–Epac(δDEPCD)–mCitrine) [12] was cloned as previously described [13] into the mammalian expression vector pSTZ-4 (STZ Angewandte Biologische Chemie, Mannheim, Germany), which contains a bidirectional tetracycline-inducible promotor where transcription is turned on in the absence of doxycycline (Tet-Off; [14]), and the neo gene that confers resistance to Geneticin (G418). The CEPAC expression vector was cotransfected into CHO-K1 cells along with the mammalian expression vector pSTZ-5 (STZ Angewandte Biologische Chemie, Mannheim, Germany) containing the hygromycin phosphotransferase gene that confers resistance to hygromycin, and the Tet-Off inducible truncated human soluble adenylate cyclase (sAC^tr^) gene coding for a truncated version of human soluble adenylate cyclase (NCBI Reference Sequence: XP_006711512.1, amino acid 1-469) lacking the large C-terminal region and resulting in a higher catalytic activity compared to the full length sAC [15,16]. In order to generate stably transfected cell lines, cells were cotransfected at a confluence of 75 % in 12-well plates. Cotransfection was conducted using FUGENE-6 (Promega, Mannheim, Germany) according to the manufacturer’s instructions. After 48 h of cultivation, cells were selected and expanded under 0.6 mg/ml G418-sulfate and 0.48 mg/ml hygromycin B. Upon induction of transgene expression in the resulting sAC-CEPAC (soluble adenylate cyclase-CEPAC cAMP FRET reporter) cells via doxycycline withdrawal from the culture medium, subclones were screened for CEPAC expression and sAC^tr^ activity using the agonist bicarbonate. Upon sAC^tr^ activation the intracellular cAMP level increases leading to an increase in the emission fluorescence ratio Em(480 nm/530 nm) when excited at 430 nm as described in reference [12].

### 2.3. Co-Expression of the sAC-CEPAC Bicarbonate Sensory Cellular Test System with CFTR

CHO-K1 cells were co-transfected with plasmids coding for CEPAC (pSTZ-4) and sAC^tr^ and CFTRwt (NCBI Reference Sequence: NP_000483.3) (pSTZ-5) at a confluence of 75 % in 12-well plates. After 48 h of cultivation, cells were selected and expanded under 0.6 mg/ml G418-sulfate and 0.48 mg/ml hygromycin B. The transfection was conducted using FUGENE-6 (Promega, Mannheim, Germany) according to the manufacturer’s instructions. Upon induction of transgene expression via doxycycline withdrawal from the culture medium, subclones were screened for CEPAC expression as well as sAC^tr^ and CFTR activity using the CFTR-specific inhibitor 172 (Sigma-Aldrich, Steinheim, Germany). Upon addition of bicarbonate to the bicarbonate-free assay buffer the sAC^tr^ is activated and the intracellular cAMP level increases leading to an increase in the fluorescence emission ratio Em(480 nm/530 nm) when excited at 430 nm. An increasing cAMP level leads to the activation of CFTR resulting in an additional increase of cAMP.

### 2.4. Bicarbonate Biosensor Assay

Cells were seeded 24h before assay in 96-wells in Ham’s F12 medium (10 % FBS). Induction of expression followed the withdrawal of doxycycline. After 21 h of induction of expression cell culture medium was replaced by bicarbonate-free HEPES buffer (25 mM HEPES pH 7.3 set with KOH, 126 mM NMDG-Cl, 5.4m M KCl, 5.4 mM glucose, 0.8m M MgCl_2_, 1.8 mM CaCl_2_), in which cells were bicarbonate-depleted to a basal level for 3 h in order to reduce the intracellular cAMP concentration to a basal level. After replacement of bicarbonate-free HEPES buffer with assay buffer (bicarbonate-free HEPES buffer containing 0.5 % DMSO), the measurement of the fluorescence emission ratio Em(480 nm/530 nm) when excited at 430 nm was taken prior to the replacement of bicarbonate-free assay buffer with assay buffer containing different dilutions of bicarbonate assay buffer (25 mM HEPES pH 7.3 set with KOH, 126 mM KHCO_3_, 5.4 mM KCl, 5.4 mM glucose, 0.8 mM MgCl_2_, 1.8 mM CaCl_2_, 0.5 % DMSO) in bicarbonate-free assay buffer. Then, the measurement of the fluorescence intensity ratio continued for further 25 min.

### 2.5. Forskolin-induced cAMP Synthesis

Cells were seeded 24h before assay in 96-wells in Ham’s F12 medium (10 % FBS). Induction of expression followed the withdrawal of doxycycline. After 21 h of induction of expression, cell culture medium was replaced by bicarbonate-free HEPES buffer (25 mM HEPES pH 7.3 set with KOH, 126 mM NMDG-Cl, 5.4 mM KCl, 5.4 mM glucose, 0.8 mM MgCl_2_, 1.8 mM CaCl_2_), in which cells were bicarbonate-depleted to a basal level for 3 h in order to reduce the intracellular cAMP concentration to a basal level. After replacement of bicarbonate-free HEPES buffer with assay buffer (bicarbonate-free HEPES buffer containing 0.5 % DMSO) the measurement of the fluorescence emission ratio Em(480 nm/530 nm) when excited at 430 nm was taken prior to the replacement of bicarbonate-free assay buffer with assay buffer containing different dilutions of forskolin (Sigma-Aldrich, Steinheim, Germany; 25 mM stock solution in DMSO) in bicarbonate-free assay buffer. Then, the measurement of the fluorescence intensity ratio continued for further 25 min.

### 2.6. Bicarbonate Influx Assay—Bicarbonate Transport Activity of Prodigiosin

Cells were seeded 24 h before assay in 96-wells in Ham’s F12 medium (10 % FBS). Induction of expression followed the withdrawal of doxycycline. After 21 h of induction of expression cell culture medium was replaced by bicarbonate-free HEPES buffer (25 mM HEPES pH 7.3 set with KOH, 126 mM NMDG-Cl, 5.4 mM KCl, 5.4 mM glucose, 0.8 mM MgCl_2_, 1.8 mM CaCl_2_), in which cells were bicarbonate-depleted to a basal level for 3h in order to reduce the intracellular cAMP concentration to a basal level. After replacement of bicarbonate-free HEPES buffer with a dilution series of prodigiosin (Roberto Quesada, University of Burgos, Burgos, Spain; 20 mM stock solution in DMSO) in assay buffer (bicarbonate-free HEPES buffer containing 0.5 % DMSO) and 10 min pre-incubation of the cells therein, the measurement of the fluorescence emission ratio Em(480 nm/530 nm) when excited at 430 nm was taken prior to the addition of bicarbonate assay buffer (25m M HEPES, 126 mM KHCO_3_ to give pH 7.3, 5.4 mM KCl, 5.4 mM glucose, 0.8 mM MgCl_2_, 1.8 mM CaCl_2_, 0.5 % DMSO) to a final bicarbonate concentration of 25.2 mM. Then, the measurement of the fluorescence intensity ratio continued for further 25 min. For the generation of prodigiosin concentration-response curves, the fluorescence emission ratio Em(480 nm/530 nm) was determined 14 min after bicarbonate addition when the percentage of fluorescence emission ratio increase relative to the DMSO control was greatest.

### 2.7. Bicarbonate Efflux Assay - Bicarbonate Transport Activity of Prodigiosin

Cells were seeded 24 h before assay in 96-wells in Ham’s F12 medium (10 % FBS). Induction of expression followed the withdrawal of doxycycline. After 23.5 h of induction of expression cell culture medium was replaced by bicarbonate assay buffer (25 mM HEPES, 126 mM NaHCO_3_ to give pH 7.3, 5.4 mM KCl, 5.4 mM glucose, 0.8 mM MgCl_2_, 1.8 mM CaCl_2_), in which cells were loaded with bicarbonate for 0.5 h in order to raise the intracellular cAMP concentration to a maximal level. After replacement of bicarbonate HEPES buffer with a dilution series of prodigiosin (Roberto Quesada, University of Burgos) in bicarbonate assay buffer (bicarbonate HEPES buffer containing 0.5 % DMSO) and 10 min pre-incubation of the cells therein, the measurement of the fluorescence emission ratio Em(480 nm/530 nm) when excited at 430 nm was taken prior to the replacement of the dilution series of prodigiosin in bicarbonate assay buffer with the same dilution series of prodigiosin in bicarbonate-free assay buffer (25 mM HEPES pH 7.3 set with KOH, 126 mM NMDG-Cl, 5.4 mM KCl, 5.4 mM glucose, 0.8 mM MgCl_2_, 1.8 mM CaCl_2_, 0.5 % DMSO). Then, the measurement of the fluorescence intensity ratio continued for further 25 min. For the generation of prodigiosin concentration–response curves, the fluorescence emission ratio Em(480 nm/530 nm) was determined 9 min after the change to bicarbonate-free buffer when the percentage of fluorescence emission ratio decrease relative to the DMSO control was greatest.

### 2.8. CFTR Biosensor Assay

The experimental conditions of the CFTR biosensor assay corresponded to those of the bicarbonate influx assay. For the above assays, measurements were carried out in hexaplicate. Background fluorescence correction for prodigiosin was carried out by subtracting the fluorescence of uninduced cells from induced cells treated with the same prodigiosin concentration. Uninduced cells remained treated with doxycycline during the assay. Fluorescence measurements were carried out with a ClarioStar plate reader (BMG Labtech, Ortenberg, Germany). ClarioStar instrument settings for the assays were excitation 430 nm, emission 480 nm, and 530 nm; plate mode.

### 2.9. BCECF Assay

CHO-K1 cells were seeded in 96-wells in Ham’s F12 medium (10 % FBS) 24 h before the assay. Culture medium was removed and cells were incubated in bicarbonate-free HEPES buffer with and without sodium ions (25 mM HEPES pH 7.3 set with NaOH, 126 mM NaCl, 5.4 mM KCl, 5.5 mM glucose, 0.8 mM MgCl_2_, 1.8 mM CaCl_2_ and 25 mM HEPES pH 7.3 set with KOH, 126 mM NMDG-Cl, 5.4 mM KCl, 5.5 mM glucose, 0.8 mM MgCl_2_, 1.8 mM CaCl_2_, respectively) for 2.5 h. Then, cells were incubated with 2 µM BCECF (BCECF/AM Sigma-Aldrich GmbH Munich, Germany) in bicarbonate-free HEPES buffer (+/− sodium ions) for 15 min. After the replacement of dye-containing buffer with bicarbonate-free HEPES buffer (+/− sodium ions), the fluorescence excitation ratio 490 nm/440 nm (emission 535 nm) was measured as described in reference [17]. Then, bicarbonate-containing buffer (+/− sodium ions) was added to a final bicarbonate concentration of 25 mM and measurement continued for further 20 min.

## 3. Results

### 3.1. Bicarbonate Biosensor Assay

**Figure 2:**
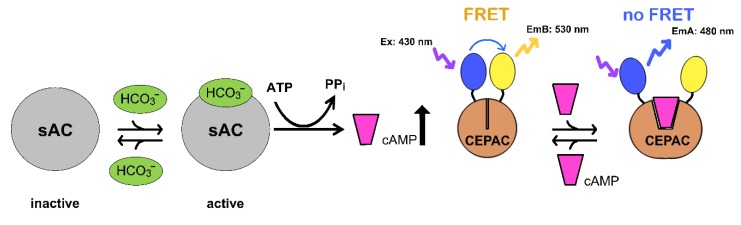
Bicarbonate sensory cellular test system. Upon induction via doxycycline withdrawal from the culture medium, the sAC-CEPAC cell line expresses the bicarbonate sensory cellular test system consisting of the catalytically active bicarbonate sensor soluble adenylate cyclase (sAC^tr^) and the cAMP reporter CEPAC. When sAC^tr^ is activated by bicarbonate (HCO_3_^−^), the enzyme catalyzes cAMP synthesis and cAMP then binds to CEPAC. This results in a reduced energy transfer from the blue to the yellow fluorophore measured as a decrease in FRET intensity. When the bicarbonate sensory cellular test system is implemented in an enzymatically coupled bicarbonate assay, an increase in the fluorescence emission ratio due to a sAC^tr^-mediated increase in cAMP serves as a measure for the increase in intracellular bicarbonate concentration.

After induction, the bicarbonate sensory cellular test system is expressed in CHO-K1 cells. It consists of a truncated catalytically active soluble adenylate cyclase (sAC^tr^) and the cAMP FRET (Förster Resonance Energy Transfer) reporter CEPAC. An increase in intracellular bicarbonate concentration activates sAC^tr^ resulting in an increase in intracellular cAMP concentration. cAMP binds to the regulatory domain of CEPAC resulting in a decrease in FRET intensity (Figure 2).

**Figure 3:**
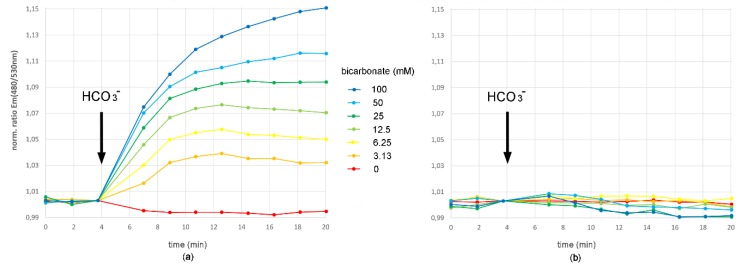
Bicarbonate biosensor assay. The bicarbonate sensory cellular test system measures bicarbonate influx in a concentration-dependent manner in a range from 0 to 100 mM bicarbonate. Upon addition of bicarbonate to the assay buffer, an intracellular increase in bicarbonate concentration resulted in an increase in the fluorescence emission ratio Em(480 nm/530 nm) when exited at 430 nm in (**a**) the CHO-K1 cell expressing both, the bicarbonate sensor sAC^tr^ and the cAMP reporter CEPAC. This ratio increase was dependent on the intracellular increase in cAMP concentration catalyzed by sAC^tr^, since (**b**) a control cell expressing only the CEPAC reporter without concomitant sAC^tr^ expression showed no bicarbonate-dependent increase in the fluorescence emission ratio upon bicarbonate addition. Data are expressed as mean (n = 6).

After pre-incubation of the sAC-CEPAC expressing cells in bicarbonate-free buffer the addition of bicarbonate led to a concentration-dependent increase in the fluorescence emission ratio Em(480 nm/530 nm), which was normalized to the emission ratio before the addition of bicarbonate (Figure 3a). The ratio increase was sAC^tr^-dependent. A control cell expressing the CEPAC reporter without concomitant sAC^tr^ expression showed no bicarbonate-dependent ratio increase under the same assay conditions (Figure 3b).

**Figure 4:**
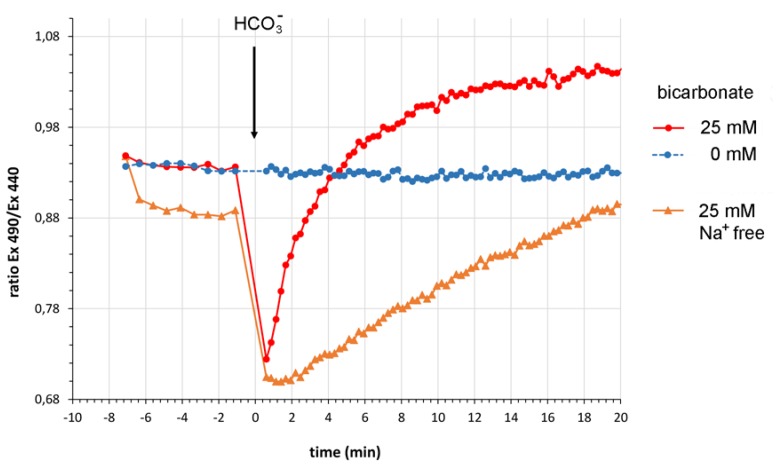
Bicarbonate-induced intracellular pH changes measured by H^+^-sensitive BCECF. Upon bicarbonate addition, after an initial pH drop most likely due to CO_2_ entering the cell, the pH increases due to bicarbonate influx into CHO-K1 cells. The effect was much more pronounced in the presence of Na^+^ suggesting the involvement of Na^+^/HCO_3_^−^ symport. Data are expressed as mean (n = 6).

This unchanged fluorescence ratio of the CEPAC reporter in the control cell proves the pH-independence of the CEPAC ratio measurement because the addition of bicarbonate to bicarbonate-free assay buffer must have resulted in intracellular pH changes as depicted in Figure 4. There, in an assay employing the pH indicator BCECF, after the addition of bicarbonate to a final concentration of 25 mM the bicarbonate transport metabolon activity of CHO-K1 cells first led to a fast pH decrease measured by a decrease in BCECF fluorescence emission ratio when excited at two different wavelengths. This initial pH decrease is most likely due to an influx of carbon dioxide, which is in equilibrium with bicarbonate and which is also produced by the activity of extracellular carbonic anhydrase. After the initial pH decrease follows an intracellular pH net increase due to cellular mechanisms. This pH increase was much stronger in the presence of Na^+^ and must, therefore, be partly the result of Na^+^-dependent bicarbonate transport mechanisms such as Na^+^/HCO_3_^−^ symport. In the bicarbonate biosensor assay, these sodium ion-dependent bicarbonate transport mechanisms were excluded by working under sodium ion-free buffer conditions where sodium chloride was replaced by NMDG-Cl.

### 3.2. Bicarbonate Biosensor Assay

**Figure 5:**
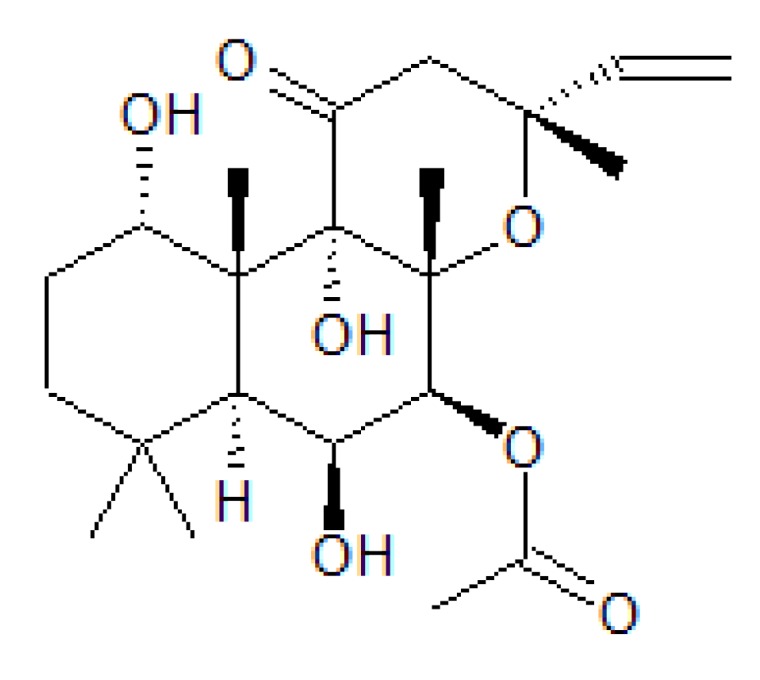
Forskolin, a labdane diterpene produced by the plant Plectranthus barbatus activates the membrane bound enzyme adenylate cyclase and increases intracellular levels of cAMP.

The CFTR anion channel requires phosphorylation by protein kinase A (PKA) to be active. PKA is activated by cAMP. Extracellular application of forskolin (Figure 5), a terpene derivative of plant origin in µM concentrations is known to activate membrane-bound adenylate cyclase (mAC) resulting in the production of sufficient cAMP to activate PKA and the subsequent activation of CFTR [18] through phosphorylation. 

**Figure 6:**
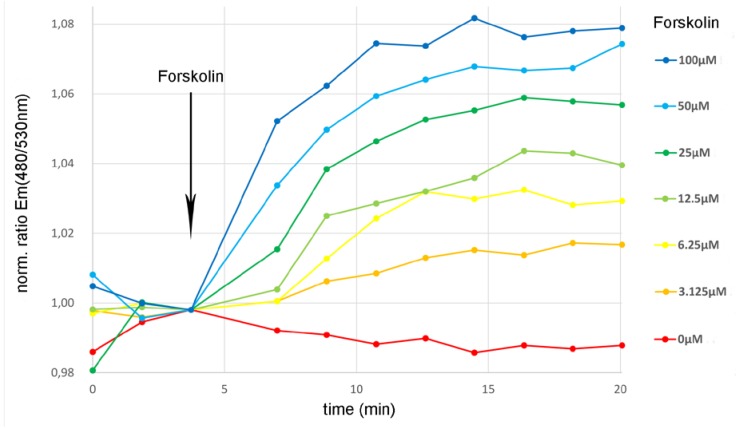
Forskolin-induced cAMP synthesis. The cAMP specificity of the CEPAC reporting unit of the bicarbonate sensory cellular test system is demonstrated by the activation of endogenous membrane-bound andenylate cyclase (mAC) by forskolin in a concentration-dependent manner in the range from 0 to 100 µM forskolin. Upon addition of forskolin to the assay buffer, activated mAC catalyzed cAMP synthesis leading to an intracellular increase in cAMP concentration. This resulted in an increase in the fluorescence emission ratio of the cAMP reporter CEPAC in the bicarbonate sensor cell. This increase in the fluorescence emission ratio elicited through the activation of mAC by forskolin was similar to the fluorescence emission ratio increase elicited through the activation of sAC^tr^ by bicarbonate (Figure 3). Data are expressed as mean (n = 6).

We compared the forskolin-induced with the bicarbonate-induced intracellular cAMP increase in cells expressing sAC^tr^ and CEPAC. We find that both cAMP increases follow similar kinetics (Figure 3a and Figure 6) and differ clearly from the intracellular bicarbonate induced pH change (Figure 4) confirming a nearly complete pH independence of the sensory cellular test system. The application of 25 µM forskolin resulted in an intracellular cAMP increase similar to the application of 25 mM bicarbonate. Therefore, it should be possible to activate CFTR in a living cell expressing sAC^tr^, CEPAC and CFTR simultaneously by adding bicarbonate to the assay buffer. To test whether the addition of bicarbonate into the assay buffer ultimately leads to an activation of CFTR via PKA-mediated phosphorylation upon PKA activation via sAC-mediated cAMP increase we developed a CFTR bicarbonate biosensor cell line expressing sAC^tr^, CEPAC and CFTR simultaneously upon induction of expression.

**Figure 7:**
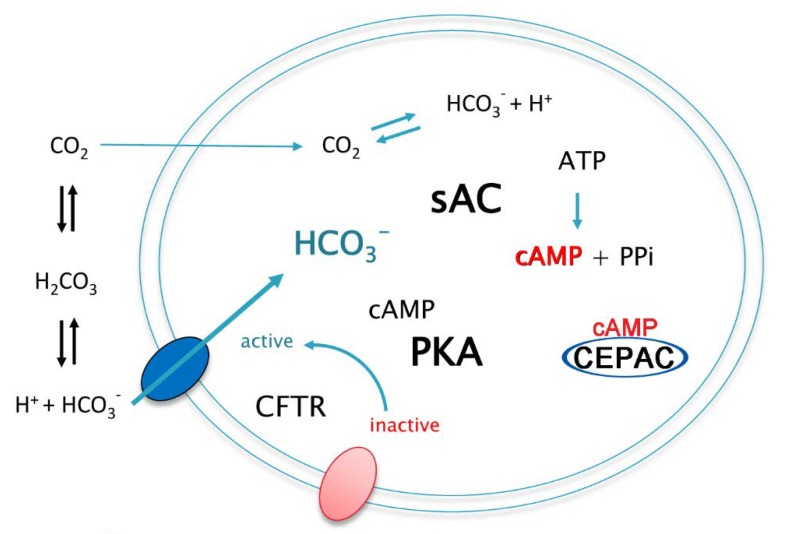
Mechanism of cystic fibrosis transmembrane conductance regulator (CFTR) activation in the sAC-CEPAC-CFTR cell. In the sAC-CEPAC-CFTR cell, CFTR is activated by PKA- (protein kinase A) dependent phosphorylation causing the channel to open. Bicarbonate influx results in cAMP synthesis catalyzed by sAC^tr^. cAMP activates PKA which in turn activates CFTR through phosphorylation.

We then added bicarbonate to the bicarbonate-free assay buffer of the sAC-CEPAC-CFTR cell line up to a final concentration of 25 mM to achieve an increase in the intracellular bicarbonate concentration as previously described for the sAC-CEPAC cell line without CFTR. The subsequent activation of sAC^tr^ and the resulting increase in intracellular cAMP concentration led to an initial increase in the fluorescence emission ratio Em(480 nm/530 nm) of the cAMP FRET reporter CEPAC. At the same time, cAMP activates PKA which in turn activates CFTR. Activated CFTR then channels bicarbonate along the gradient from the outside to the inside of the cell (Figure 7). This results in an additional increase of intracellular bicarbonate concentration and a concomitant increase of the fluorescence emission ratio Em(480 nm/ 530 nm).

**Figure 8:**
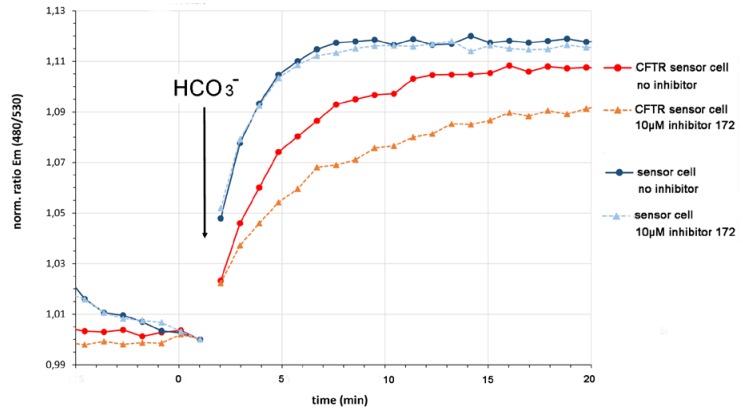
CFTR bicarbonate transport activity. Bicarbonate addition resulted in a CFTR-dependent intracellular increase in cAMP. CFTR inhibition with 10 µM CFTR-specific inhibitor 172 resulted in a reduced cAMP-dependent fluorescence emission ratio increase due to a diminished bicarbonate influx (orange) compared to the control with no CFTR inhibitor present (red). The bicarbonate sensor cell expressing no CFTR served as a control and showed no CFTR inhibitor-dependent difference in cAMP concentration increase due to an inhibitor-independent bicarbonate influx (blue). The higher response to bicarbonate of the cell without CFTR compared to the cell with CFTR is explained most likely by higher levels of sAC^tr^ and CEPAC when a cell has to make only two compared to three proteins upon induction. Data are expressed as mean (n = 6).

To distinguish between the CFTR activity and other cellular bicarbonate transport activities we pre-incubated the sAC-CEPAC-CFTR cell with 10 µm of the CFTR-specific inhibitor 172 in sodium ion-free buffer. NMDG-Cl replaced sodium chloride in the buffer medium. Under these experimental conditions, CFTR inhibition resulted in a reduced cAMP-dependent fluorescence emission ratio increase due to a diminished bicarbonate influx (Figure 8, orange) compared to the control with no CFTR inhibitor present (red). The sAC-CEPAC cell expressing no CFTR served as a control and showed no CFTR inhibitor-dependent difference in cAMP concentration increase, thereby confirming the CFTR-specificity of the inhibitor 172 (Figure 8. blue). 

The higher response of the sAC-CEPAC cell to bicarbonate compared to the sAC-CEPAC-CFTR cell is explained by higher levels of the sensing (sAC^tr^) and reporting unit (CEPAC) of the bicarbonate sensory cellular test system in the cell that does not have to make the additional CFTR protein upon induction.

### 3.3. Bicarbonate Transport Activity of the Natural Anionophore Prodigiosin

**Figure 9:**
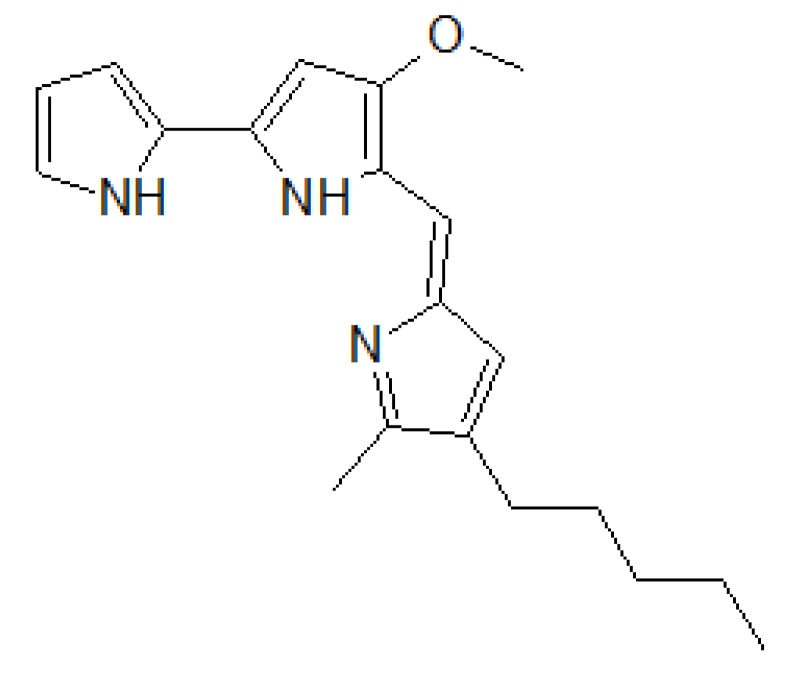
Prodigiosin is a secondary metabolite of the bacterium Serratia marcescens. The anionophore prodigiosin transports chloride and bicarbonate ions through biological membranes of living cells.

The prodiginines are alkaloids belonging to a family of compounds produced by several bacteria and are well known for their anionophoric activity [19]. Prodigiosin (Figure 9) is the most studied natural anionophore facilitating chloride and bicarbonate transport across biological membranes [20].

**Figure 10:**
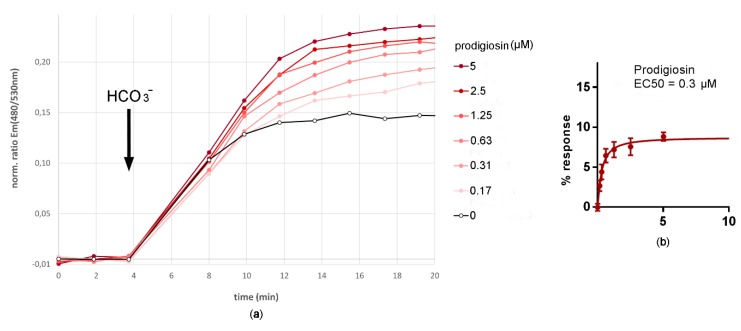
Bicarbonate influx assay—bicarbonate transport activity of prodigiosin. The bicarbonate biosensor cell line measures bicarbonate influx facilitated by the naturally occurring anionophore prodigiosin in a concentration-dependent manner in a range from 0 to 5 µM prodigiosin: Upon addition of 25 µM bicarbonate to the assay buffer, an intracellular increase in bicarbonate concentration facilitated by prodigiosin caused an intracellular increase in cAMP catalyzed by sAC^tr^. (**a**) This resulted in an increase in the fluorescence emission ratio Em(480 nm/530 nm) when exited at 430 nm in the bicarbonate sensor cell expressing both, the sensory unit sAC^tr^ and the reporting unit CEPAC of the bicarbonate sensory cellular test system. (**b**) A 4-parameter sigmoid concentration-response curve was generated from the percentages of changes in fluorescence emission ratio relative to the control without prodigiosin 14 min after bicarbonate addition. From this concentration-response curve, the EC50 value for prodigiosin was calculated to be 0.3 µM. Data are expressed as mean (n = 6). Error bars represent the mean standard deviation.

Bicarbonate influx mediated by prodigiosin was measured using the developed bicarbonate sensor cell. After pre-incubation of the sensor cell in sodium ion and bicarbonate-free buffer with different prodigiosin concentrations, bicarbonate addition resulted in a concentration-dependent increase of the cAMP-dependent fluorescence ratio. The kinetic response, as well as the concentration-response curve are shown in Figure 10. The EC50 concentration for prodigiosin under these conditions was calculated to be 0.3 µM.

**Figure 11:**
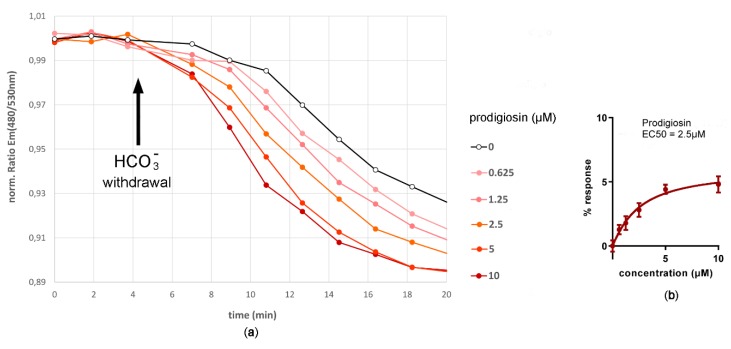
Bicarbonate efflux assay - bicarbonate transport activity of prodigiosin. The bicarbonate biosensor cell line measures bicarbonate efflux facilitated by the naturally occurring anionophore prodigiosin in a concentration-dependent manner in a range from 0 to 10 µM prodigiosin. Upon withdrawal of bicarbonate from the assay buffer, an intracellular decrease in bicarbonate concentration caused an intracellular decrease in cAMP. (**a**) This resulted in a decrease in the fluorescence emission ratio Em(480 nm/530 nm) when exited at 430 nm in the bicarbonate sensor cell expressing both, the sensory unit sAC^tr^ and the reporting unit CEPAC of the bicarbonate sensory cellular test system. (**b**) A 4-parameter sigmoid concentration-response curve was generated from the percentages of changes in fluorescence emission ratio relative to the control without prodigiosin 9 min after bicarbonate withdrawal. From this concentration-response curve, the EC50 value for prodigiosin was calculated to be 2.5 µM. Data are expressed as mean (n = 6). Error bars represent the mean standard deviation.

In addition, bicarbonate efflux induced by prodigiosin was also measured using the developed bicarbonate sensor cell. After pre-incubation of the sensor cell in high bicarbonate buffer with different prodigiosin concentrations, the replacement of high bicarbonate buffer with bicarbonate-free buffer resulted in a concentration-dependent decrease of the cAMP-dependent fluorescence ratio due to a decrease in intracellular bicarbonate concentration. The kinetic response, as well as the concentration-response curve, are shown in Figure 11. Under these conditions, the EC50 concentration for prodigiosin was calculated to be 2.5 µM. This higher EC50 value in the bicarbonate efflux assay compared to 0.3 µM in the bicarbonate influx assay is most likely explained by a strong intracellular pH increase during loading of the cell with 126 mM bicarbonate exceeding the extracellular pH of 7.3 and, as a consequence, prodigiosin being more frequently deprotonated and therefore less active as anion transporter at the highly alkaline intracellular pH.

## 4. Discussion

The role of bicarbonate is of great significance to human physiology ranging from cellular respiration to pH homeostasis. Bicarbonate movements across biological membranes influence the intracellular pH and can induce cellular responses that depend on changes in intracellular bicarbonate concentration and/or changes in intracellular pH. It is therefore of critical importance that bicarbonate transport across cell membranes is tightly regulated. However, despite the enormous importance of bicarbonate and bicarbonate transport at the cellular level, measurement of bicarbonate concentration changes in living cells, so far, has only been possible by measuring intracellular pH changes, since, until today, no bicarbonate-specific pH-independent sensor system has been described.

In this work, we are describing a novel genetically encoded, fluorescence-based bicarbonate sensory cellular test system for monitoring bicarbonate concentration changes in living cells in a pH-independent manner. The developed bicarbonate sensory cellular test system consists of a truncated catalytically active soluble adenylate cyclase sAC^tr^ that serves as the bicarbonate sensory unit catalyzing the bicarbonate concentration-dependent synthesis of cAMP, and of the reporting unit CEPAC. cAMP binds to the regulatory domain of CEPAC resulting in a cAMP concentration-dependent FRET intensity decrease. The bicarbonate sensor cell expressing sAC^tr^ and CEPAC simultaneously allows for a real-time readout of intracellular bicarbonate-dependent cAMP concentration changes.

Along with this bicarbonate sensory cellular test system, a cellular assay was developed for the characterization of the bicarbonate transport activity of anionophores, such as prodigiosin, in a microplate format. Anionophores are small molecules capable of facilitating the transmembrane transport of anions. After establishing a bicarbonate biosensor cell line expressing the sensor sAC^tr^ and the reporter CEPAC simultaneously, the major challenge was the development of assay conditions under which the characterization of anionophore-specific bicarbonate transport activity against the background of cellular bicarbonate transport became feasible. Eventually, cell incubation in sodium ion- and bicarbonate-free buffer at 22–25 °C and the addition of bicarbonate to a final concentration of 25.2 µM upon 10 min pre-incubation of the cells with the anionophore prodigiosin was found to be suitable for the characterization of the concentration-dependent bicarbonate transport activity of prodigiosin in the bicarbonate influx assay.

Conversely, cell incubation in 126 mM sodium bicarbonate buffer at 22–25 °C and the replacement of 126 mM sodium bicarbonate buffer with sodium ion- and bicarbonate-free buffer upon 10 min pre-incubation of the cells with the anionophore prodigiosin was found to be an appropriate setting for the concentration-dependent bicarbonate transport activity measurement of prodigiosin in the bicarbonate efflux assay. However, under these assay conditions during bicarbonate efflux, it must be taken into consideration that the initial high intracellular pH due to pre-incubation of the cells in 126 mM bicarbonate decreases after changing to 0 mM bicarbonate due to prodigiosin-mediated efflux. Therefore, even though the bicarbonate sensory cellular test system itself does measure intracellular bicarbonate concentration in a pH-independent manner, the known diminished anion transport activity of prodigiosin at high pH is most likely responsible for the lower potency of prodigiosin observed in the bicarbonate efflux assay compared to the bicarbonate influx assay.

In addition, under the chosen bicarbonate influx assay conditions we measured the bicarbonate transport activity of wild type human CFTR, a bicarbonate- (and chloride) conducting transmembrane channel protein, using a bicarbonate sensor cell line which co-expressed CFTR with the sensory unit sAC^tr^ and the reporting unit CEPAC of the bicarbonate sensory cellular test system. CFTR-mediated bicarbonate influx was verified by implementing the CFTR-specific inhibitor 172 in the assay, by which bicarbonate influx was diminished. Accordingly, as expected, the CFTR-specific inhibitor 172 was found to have no effect on bicarbonate influx in control cells expressing sAC^tr^ and CEPAC only.

In summary, the usefulness of the bicarbonate sensory cellular test system has been successfully tested by employing the naturally occurring and well-studied anionophore prodigiosin in measurements of prodigiosin-mediated bicarbonate concentration changes in living cells. Anionophores replacing the defective CFTR activity in cystic fibrosis patients represent promising drug candidates for CF therapy [21]. The bicarbonate sensory cellular test system could be used in screening of novel anionophore library compounds for bicarbonate transport activity with efficiencies close to the natural anion channel CFTR. The bicarbonate sensory cellular test system was found to be useful in measuring intracellular bicarbonate concentration changes in response to activation and specific inhibition of wild type human CFTR protein when co-expressed with sAC^tr^ and CEPAC in living cells. This underscores the valuable benefit of this novel bicarbonate sensory cellular test system in studying the functionality of bicarbonate transporting proteins in response to activation and inhibition when co-expressed in living cells and employed in bicarbonate influx or efflux assays.

## 5. Conclusions

Bicarbonate plays a central role in human physiology, and defective bicarbonate transport results in several diseases. In this study, we report the development of a novel genetically encoded fluorescence-based bicarbonate sensory cellular test system for monitoring intracellular bicarbonate concentration changes in living cells in a pH-independent manner. We show that the developed system is suitable for the characterization of bicarbonate transport activity of anionophore compounds in living cells. Further, we demonstrate the usefulness of the developed system in studying the functionality of the bicarbonate transport protein CFTR.
